# Biochar Blended with Nitrogen Fertilizer Promotes Maize Yield by Altering Soil Enzyme Activities and Organic Carbon Content in Black Soil

**DOI:** 10.3390/ijerph20064939

**Published:** 2023-03-10

**Authors:** Jing Sun, Xinrui Lu, Shuang Wang, Chunjie Tian, Guoshuang Chen, Nana Luo, Qilin Zhang, Xiujun Li

**Affiliations:** 1 Key Laboratory of Wetland Ecology and Environment, Northeast Institute of Geography and Agroecology, Chinese Academy of Sciences, Changchun 130102, China; 2 University of Chinese Academy of Sciences, Beijing 100049, China; 3 Affairs Service Centers of Natural Resources in Tieling Country, Northeast China, Tieling 112608, China

**Keywords:** biochar, organic carbon, enzyme activity, maize yield, black soil

## Abstract

Biochar and nitrogen fertilizers are known to increase soil carbon storage and reduce soil nitrogen loss as amendments, suggesting a promising strategy for highly effectively increasing soil productivity. However, few studies have explored the mechanisms of their effects on crop yield in terms of active carbon fraction and enzyme activity, which ultimately limits the potential for the application of biochar in combination with nitrogen fertilizers. To evaluate the effect of biochar and nitrogen fertilizer on the improvement of black soils in northeast China, a field experiment was conducted in the black soil to compare and analyze the application methods on total organic carbon (TOC), total nitrogen (TN), enzyme activities, and maize yields. Biochar rates: CK, C1, C2, and C3 (0, 9.8, 19.6, and 29.4 Mg·ha^−1^); N fertilizer rates: N1/2 and N (30 and 60 kg·ha^−1^). Results indicated that biochar and N fertilizer amendments significantly ameliorated soil fertility, such as TOC and TN, compared to the unamended soil. The TOC levels in the C3 treatment increased by 35.18% and the TN levels by 23.95%. The improvement in TN is more significant when biochar is blended with N fertilizer. Biochar blended with N fertilizer increased maize cellulase, urease, and invertase activities by an average of 53.12%, 58.13%, and 16.54%, respectively. Redundancy analysis showed that TOC, TN, and MBN contributed 42%, 16.2%, and 22.2%, respectively, to the maize yield indicator. Principal component analysis showed that reduced N fertilizer was more effective in improving yields, with a maximum yield increase of 50.74%. Biochar blended with N fertilizer is an effective strategy to improve the fertility and productivity of black soils in northeast China, while nitrogen fertilizer reduction is feasible and necessary for maintaining grain yield.

## 1. Introduction

Biochar is considered a solution for improving the soil’s physical structure, forming a highly stable carbon pool, and regulating global climate change [1,2]. The beneficial effects of biochar on soil fertility have been explored in the Brazilian Amazon since pre-Columbian times; most soils in the region are highly weathered and depleted oxidized soils [3]. These modified Terra Preta soils were characterized by 35% black carbon in the surface layer [4]. Additionally, Hu [5] proposed that the mineralization of soil organic carbon (SOC) showed a strong negative excitation effect following the biochar addition, which reduced the mineralization rate of the SOC. Song [6] also indicated that the addition of biochar could increase the content of the SOC, which could be used by microorganisms, thus inhibiting organic carbon mineralization. Furthermore, Luo [7] found that exogenous carbon input can improve soil cohesion, prevent soil erosion, and decrease soil nutrient loss. Compared with the SOC, active organic carbon components (extractable organic carbon (EOC), dissolved organic carbon (DOC), and microbial biomass carbon (MBC)) respond quickly to the soil’s external environment and play an essential part in the short-term turnover of soil nutrients. They can be used as an index of early soil productivity [7,8]. EOC and DOC are characterized by high solubility, rapid mobility, easy mineralization, and decomposition [9], and their leaching or oxidative decomposition is the main pathway of organic carbon loss [10]. The effects of biochar application on soil active organic carbon fractions, microbial biomass carbon (MBC), and microbial biomass nitrogen (MBN) can reflect soil carbon accumulation and the intensity of microbial metabolism at the present stage [8]. Pan [11] explored how adding biochar and a carbon-based fertilizer affected the organic carbon pool in farmland soil. They found that the amount of highly active organic carbon was closely linked to the stability of the aggregates. The activity of soil enzymes is an important indicator of soil quality [12]. Song [6] suggested that the key factors leading to increased soil enzyme activity are improvements in the physical structure of the soil and the nutrients and other compounds contained in the biochar. Subsequently, Aziz et al. [13] proposed that fertilizers provide a large amount of unstable carbon to the soil, which microorganisms can use as a substrate food source, thereby increasing dehydrogenase activity and degradation capacity. The adsorption of biochar facilitates enzymatic reactions, as confirmed by Amoakwah et al. in a field trial of biochar-amended sandy loam soils, where the application of biochar (30 t·ha^−1^) significantly increased soil urease activity by 1.5 times and dehydrogenase activity by 3.2 times. The enzyme activity increased gradually with the increase in soil organic carbon content, further promoting the soil nutrient conversion cycle [14]. Ali [15] concluded through pot experiments that the application of nitrogen fertilizer promoted the nitrogen uptake of crops, which not only improved the photosynthetic activity of crops but also prolonged the vegetative growth period of crops. Mete [16] found through pot experiments that biochar combined with nitrogen fertilizer could increase the total biomass and seed yield of crops in alkaline soil by 391% and 367%, respectively. This also confirmed the synergistic effect of biochar and fertilizer on soybean biomass and grain yield. The basic purpose of the rational addition of biochar and N fertilizer is to reduce N loss, improve N use efficiency, and achieve the maximum benefit from the application [17,18].

The nature of biochar depends largely on the raw material and how it is applied. Since straw biochar contains fewer minerals, it should be combined with other fertilizers as much as possible in soil improvement to fundamentally increase the fertility of the soil [19,20,21,22]. Recently, there have been many studies on the application impacts of biochar in soil [8,23,24], but field investigations on soil degradation in the black soil region of northeast China were not extensive enough. There is a lack of in-depth research on the mechanism of soil active organic carbon components on yield. This paper explores the contribution of organic carbon and nitrogen, their active fractions, and enzymatic activity, respectively, to crop yield through statistical analysis. At the same time, the data support for the combined application of biochar and nitrogen fertilizer was insufficient, which limited the depth of production practice and theory [25]. We hypothesized that the combined application of biochar and nitrogen fertilizer would perform better than the application of biochar alone in increasing nitrogen content and enzyme activities. It provides a data reference for the rational allocation of biochar as nitrogen fertilizer.

The objectives of our study were (1) to explore what the effects of long-term biochar use are on soil carbon; (2) to verify the response of soil active carbon and nitrogen components and enzyme activity to combined fertilizer applications; and (3) to clarify the optimal biochar and nitrogen fertilizer rationing strategy for black soil fertility and productivity. The study provides a reference for the management of the quality of cultivated soils, the rational application of fertilizers, and the sustainable development of agriculture.

## 2. Materials and Methods

### 2.1. Site Description

The field study was conducted at the Northeast Institute of Geography and Agroecology, CAS, in Jilin Province, China (43°59′51″ N, 125°24′5″ E), 200 m above sea level, in a temperate continental climate characterized by monsoons with an annual average precipitation of 520 mm. The experimental surface soil pH was 6.06, TN was 1.26 g·kg^−1^, available phosphorus was 26.78 mg·kg^−1^, available potassium was 133.54 mg·kg^−1^, and organic matter was 26.72 g·kg^−1^. For many years, continuous corn cropping has been carried out in conventional tillage patterns. The biochar was derived from corn husk pyrolysis at 400–500 °C for 4 h under anaerobic conditions. The mean particle diameter, surface area per volume, and ash concentration of the biochar were 0.0003–3.5 mm, 0.7 m^2^·g^−1^, and 45%, respectively. The biochar had a pH of 9.16 (biochar to water of 1:10), a total carbon content of 62.64%, and a C/N ratio of 39.08. In addition, the N fertilizer was high-quality urea and was produced by Erdos Yi Ding Ecological Agriculture Development Co., Ltd., with a TN ≥ 46% and a particle size range of 1.18–3.35 mm.

### 2.2. Field Experimental Design

The biochar and N fertilizer were applied to the black soil in April 2021, and the corn was sown in May 2021. The field experiment had a split zone design with four biochar input gradients: 0 Mg·ha^−1^ (CK, 0%), 9.8 Mg·ha^−1^ (C1, 0.4%), 19.6 Mg·ha^−1^ (C2, 0.75%), and 29.4 Mg·ha^−1^ (C3, 1.5%). Also, N was applied in the form of basal fertilizer at rates of 300 kg N ha^−1^ (N1/2) and 600 kg N ha^−1^ (N) (Table 1). Each treatment was applied to a plot of 3.9 × 6.5 m^2^ and was separated by a row to avoid contamination. There were three individual treatment plots per treatment, and they were organized in a randomized block design.

### 2.3. Sample Collection and Determination

The soil samples were collected after the corn harvest was completed on 29 October 2021, and the “five-point sampling method” was used to sample 1 kg of soil in each plot. Soil drills were used to collect the 0–20 cm surface soil, and it was put into self-sealing bags. The animal and plant residues were removed, and the soil samples were immediately stored at 4 °C until the determination of the soil MBC, MBN, and enzyme activity [26]. Then, part of the soil was naturally air-dried, and the undisturbed soil was wet sieved to determine the soil aggregates. Another part of the soil samples was screened using a 2 mm screen to test the physical and chemical soil properties [27].

The potassium dichromate oxidation-external heat approach was used to determine the soil TOC [28]. Then, the TN and MBN contents were measured using the Kjeldahl method [29]. Also, the DOC, EOC, and MBC were determined by UP water concussion extraction (TOC), 333 mmol·L^−1^ potassium sulfate oxidation-colorimetry, and chloroform fumigation-K_2_SO_4_ extraction [26].

The soil invertase activity was measured using the 3,5-dinitrosalicylic acid colorimetric method [25,30]: The 5 g of soil sample was weighed and added to a 50 mL triangular flask. Then 15 mL of sucrose solution at a concentration of 8%, 5 mL of phosphate buffer (pH 5.5), and 5 drops of toluene were added to the triangular flask. Mixed, shaken thoroughly on a shaker, and incubated at 37 °C in a thermostat for 24 h. After incubation, the sample was placed in a centrifuge, centrifuged, and filtered; 1 mL of the upper filtrate was placed in a 50-mL volumetric flask, and 3 mL of the reducing agent 3,5-dinitrosalicylic acid was added to the filtrate. Placed in a thermostatic water bath and heated at 100 °C for 5 min, removed, and cooled for 3 min, the filtrate was fixed to 50 mL with distilled water and determined colorimetrically at 508 nm using a UV-Vis spectrophotometer. Cellulase activity was measured using the 3,5-dinitrosalicylic acid colorimetric method. The 10 g of soil sample was weighed, added to a 50 mL triangular flask, and then 5 mL of 1% carboxymethyl cellulose solution, 5 mL of phosphate buffer (pH 5.5), and 5 drops of toluene were added. After mixing, shake well on a shaker and incubate at 37 °C for 72 h in a thermostat. At the end of the incubation, 1 mL of the filtrate was filtered and measured colorimetrically at 549 nm using a UV-Vis spectrophotometer [31]. Urease activity was determined for each sample by measuring the formation of the product, NH4^+^ [32]: The 5 g of soil sample was weighed and added to a 50 mL triangular flask. 1 mL of toluene was added to the mouth of the flask and shaken gently for 15 min. 5 mL of 10% urea and 10 mL of citrate buffer pH 6.7 were added to the flask and mixed, then incubated at 37 °C for 2 h. After incubation, the sample was diluted to the mark with distilled water at 38 °C, shaken, and filtered. Dilute 1 mL in a 50-mL volumetric flask with distilled water to 10 mL, add 4 mL of sodium phenol solution, and 3 mL of sodium hypochlorite solution, and allow to settle to the mark after 20 min. The absorbance value was measured at a wavelength of 578 nm.

After harvest, a representative 10 m^2^ sample area was taken back to the laboratory to measure the yield; the yield of 14% water content was converted, and the spike length, spike weight, grain number per spike, and 100-grain weight and yield of each pot were statistically analyzed [33].

### 2.4. Statistical Analysis

IBM Statistics SPSS 22.0 software was used to test data normality, homogeneity, and principal component analysis (PCA). The ANOVA was performed to determine the significant differences between treatments in R (*p* < 0.05). If the data did not meet the criteria, a nonparametric Kruskal-Wallis test was performed to determine statistical significance. Fitting and mapping were done with Origin Pro 9.0.

## 3. Results

### 3.1. Soil Organic Carbon and Nitrogen Contents

**Figure 1:**
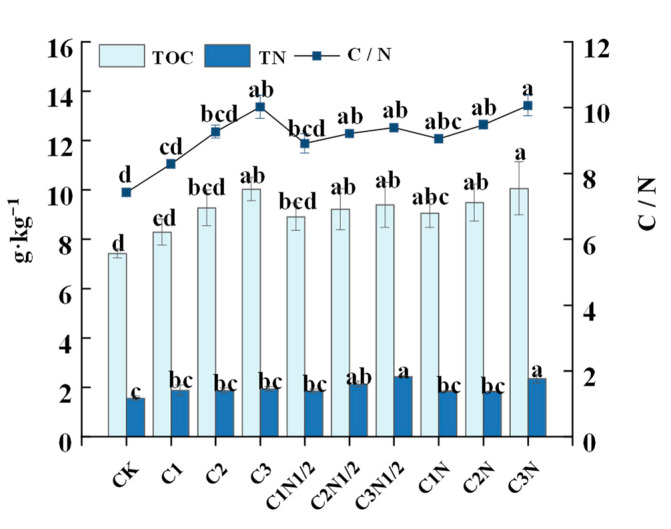
Total organic carbon (TOC), total nitrogen (TN), and carbon to nitrogen ratio (C/N) in black soils under different treatments. The bars reflect the mean standard deviation (*n* = 3). Different letters above the bars show statistically significant (*p* < 0.05) differences between the treatments.

After continuous cultivation, the initial soil (CK treatment) had no external carbon input, the soil fertility was lacking, and at this time the TOC content was 7.42 g·kg^−1^. The TOC level in the C3 treatment was 35.18% greater than the control. There was a significant positive correlation between biochar addition and soil TOC level (*p* < 0.05) (Figure 1). There was no significant difference between a combined application and a single application of TOC. Under the C3 treatment, the soil’s TN content reached 1.93 g·kg^−1^, which was 23.95% greater than that of the CK treatment. The TN content of the C3N1/2 treatment increased to 2.44 g·kg^−1^, which was 56.18% greater than that of the CK. The C/N ratio followed the same trend as TOC, peaking at C3 and C3N.

### 3.2. Soil Active Organic Carbon Components

The dynamic changes in the soil’s active organic carbon fractions are shown in Table 2. The effect of the single addition of biochar on the EOC level was not significant (*p* > 0.05). The EOC level in the bulk soil under the C3N1/2 treatment was remarkably elevated by 31.71% relative to the CK treatment and increased by 28.41% relative to the C3N treatment. Biochar increased the soil DOC content by 7.70% on average. Among the dynamic changes in the MBC, the C3 and C3N1/2 treatments had the best effect (*p* < 0.05), which were 42.09% and 44.65% greater than the CK treatment, respectively. The combined application of biochar and full nitrogen fertilizer had unsatisfactory improvement effects on MBC and MBN, with an average reduction of 12.50% and 44.62%. Furthermore, for the soil active organic carbon fraction, EOC was the main soil component, accounting for 23.69–62.91% of TOC, followed by DOC and MBC, accounting for 1.45–2.60% and 1.04–2.93% of TOC, respectively (Table 3).

### 3.3. Enzyme Activity

**Figure 2:**
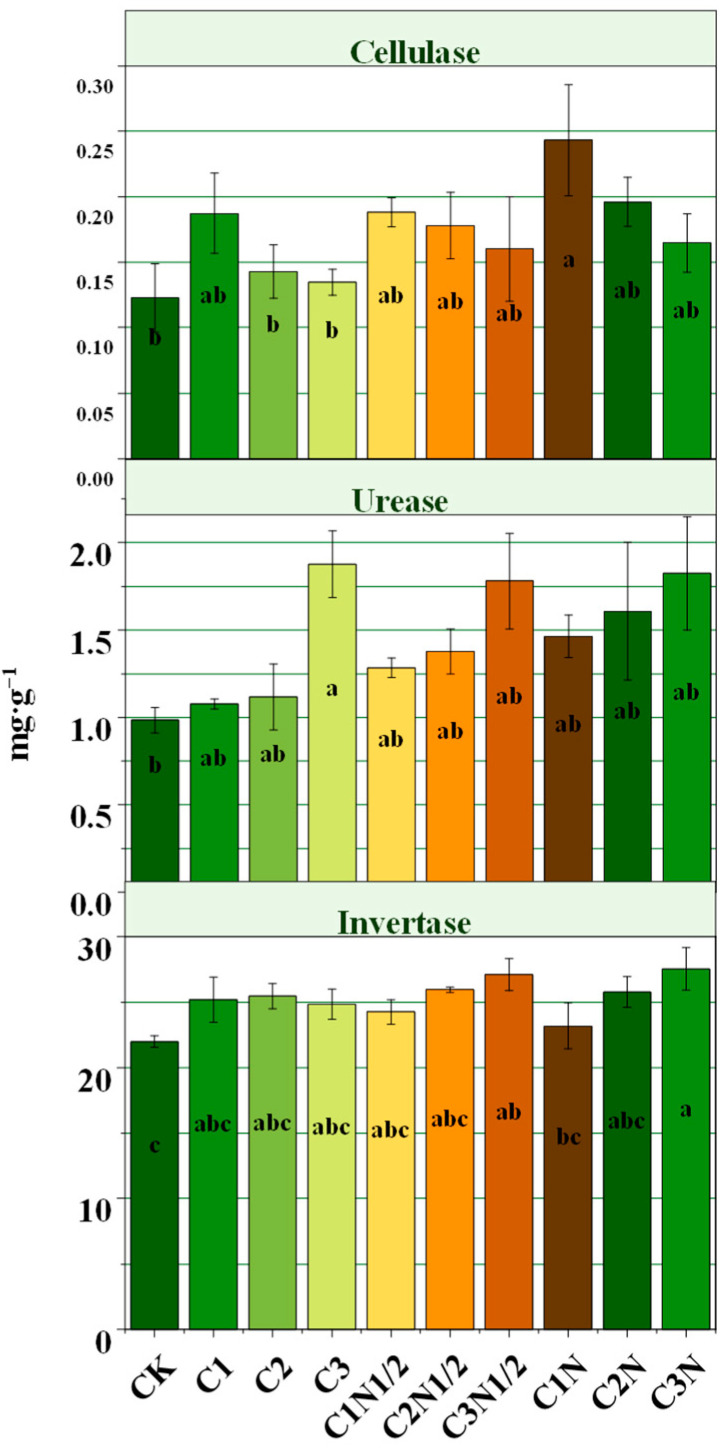
Soil enzyme activity under different treatments. Different letters indicate statistically significant (*p* < 0.05) differences between treatments.

The application of biochar and nitrogen fertilizer considerably raised the soil enzyme activity (Figure 2). The C1N treatment had the best effect, which increased cellulase activity by 98.29%. Biochar significantly increased urease activity, with an average increase of 51.36%. Biochar blended with N fertilizer was more effective in enhancing urease and cellulose. Soil convertase activity was highest in black soils, with biochar and nitrogen fertilizer increasing convertase activity by 5.43–25.23%.

**Figure 3:**
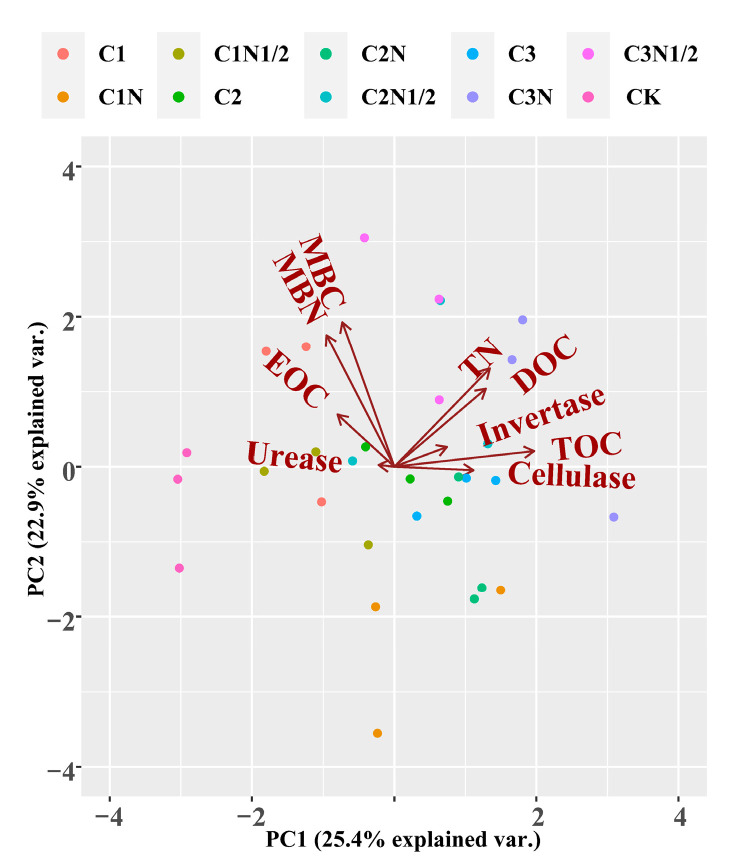
Principal component analysis of soil enzyme activity and soil carbon and nitrogen fractions.

The PCA method was used to visualize and analyze the effect of soil carbon and nitrogen fractions on enzyme activity under different treatment conditions (Figure 3). The first and second principal components (PC1 and PC2) accounted for 28.3% and 17.4% of the variance, respectively, and the treatments were significantly separated from the CK treatment. The principal component analysis (PCA) showed that biochar with nitrogen fertilizer differed significantly from the CK treatment and had the best improvement effect. There is a positive correlation between TN, TOC, and DOC and between cellulase and sucrase.

### 3.4. The Effects of the Biochar and Nitrogen Fertilizer Treatments on Maize Yield

**Figure 4:**
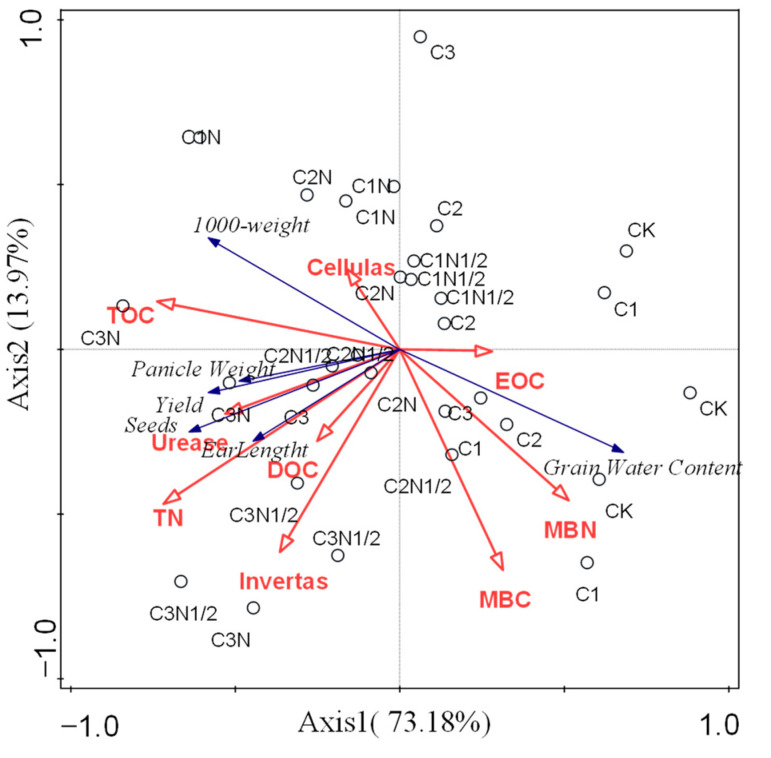
Redundancy analysis (RDA) between soil carbon and nitrogen content, their active fractions, enzyme activity, and wheat yield indicators. The blue arrows represent soil environmental factors and the red arrows represent agronomic traits of the wheat.

The findings demonstrated that the quantity of biochar had a favorable impact on the ear length, ear weight, quality, and yield of the maize. The C3 treatment increased the corn yield by 22.91%. Furthermore, the effect of the C3N1/2 treatment was the best, and the yield reached 14,052.62 kg·hm^2^ and increased by 50.97%. The yield difference was not obvious due to the difference in the N application rate (Table 4). RDA analysis showed the response of maize yield to soil environmental factors (Figure 4). TOC, TN, and MBN were significantly correlated (*p* < 0.05) with maize yield indicators, with contributions of 42%, 16.2%, and 22.2%, respectively. The contributions of sucrase, cellulase, and urease were 6.8%, 4.4%, and 3.7%, respectively.

The PCA was used to evaluate the effects of different treatments on black soil traits and yields in northeast China (Table 5). The results display that the cumulative variance contribution rate was 88.04%, which can explain the variation well. The higher the F value, the better the improvement effect, and the C3N1/2 treatment was optimal.

The expression of the principal component is:

(1) F1=0.21X1+0.40X2+0.09X3+0.03X4+0.45X5+0.39X6+0.00X7+0.39X8+0.44X9+0.30X10

(2) F2=0.44X1−0.01X2+0.51X3+0.52X4+0.09X5−0.22X6−0.37X7−0.20X8+0.06X9−0.18X10

(3) F3=0.27X1−0.37X2+0.15X3+0.06X4+0.17X5−0.21X6+0.54X7−0.14X8−0.10X9+0.61X13

where X1–X14 represent EOC, DOC, MBC, MBN, TN, TOC, cellulase activity, urea activity, invertase activity, and yield, respectively.

(4) $$F=\left(43.80\%/88.04\%\right)\times F1+\left(33.64\%/88.04\%\right)\times F2+\left(10.60\%/88.04\%\right)\times F3$$

## 4. Discussion

### 4.1. The Biochar Effect on the Soil TOC and TN

The carbon and nitrogen content of the soil is an indicator of the strength of the soil’s nutrient supply, and both biochar and N fertilizer applications are effective in increasing the medium carbon and nitrogen stocks. The results are in line with the findings by Zhang et al. (2018), showing that biochar application increased the TOC content in calcareous soils [34]. The reason for these is that biochar is a stable carbon-rich product with a carbon content of approximately 60% produced by pyrolysis of biomass under conditions of oxygen deprivation or limited oxygen supply [35]. Soil organic matter acts as a cementing substance to promote the formation of soil agglomerates, and good agglomerates promote the storage of soil organic carbon [36]. Biochar can enhance the ability of soil water and fertilizer conservation and inhibit the leaching of soil carbon and N [15]. Shi et al. proposed that the cumulative mineralization rate of TOC was reduced by 0.6–1.1% after biochar was applied with nitrogen fertilizer in loamy rice soils, which facilitated carbon aggregation and fixation [18]. In addition, Lu [37] suggested that biochar has a negative stimulatory effect on soil carbon mineralization due to the toxic substances released by biochar that may inhibit microbial activity or the preferential use of exogenous nutrients such as C and N by microorganisms, which leads to soil carbon sequestration. In this experiment, biochar and nitrogen fertilizer were applied to the soil in May. This was followed by a gradual increase in temperature and an average annual precipitation of 520 mm, concentrated in the months of June to August. The increase in soil fertility also stems from the response of urease and sucrase activity to changes in temperature and humidity, with warm and moist soil conditions favoring increased soil enzyme activity and facilitating the humification process [38]. The addition of N fertilizer alleviated the carbon limitation of soil microorganisms, supplied sufficient nutrients to plants, and increased the input of above-ground apoplankton and dead roots while improving crop yields. The results of this study showed that biochar applied with total N fertilizer (C2N and C3N treatments) reduced soil MBC and MBN content, and soil microbial biomass turnover was significantly reduced at high N addition. This may be explained by the fact that soil acidification, reduced cations, and inhibition of extracellular enzyme activity caused by N addition reduce microbial biomass, which in turn leads to reduced carbon capture and use by microorganisms [39]. According to Devereux [40], biochar has a high porosity, a hydrophilic structure, and a larger specific surface area, allowing it to retain more water and supply it to microorganisms and crops. Additionally, the role of N fertilizer is to improve the soil nutrient content, promote crop growth, increase the crop biomass, increase the return of the crop stubble and root exudates, and improve the soil TOC [12]. Song [6] discovered that the synergistic interaction between biochar and N fertilizer could significantly improve the carbon and N levels, water retention capacity, and soil nutrient availability in alkaline calcareous soils. However, it had no significant effect on the C/N or MBN. In comparison to their findings, our findings clearly showed that biochar-amended soil, particularly at higher rates (C3) or for biochar mixed with N fertilizer (C3N1/2, C3N), significantly improved the soil C/N (*p* < 0.05).

### 4.2. The Biochar Effect on the Carbon Fractions

The DOC and EOC contents, which are both useful indicators of the quality and function of the soil, reflect the capacity for the decomposition of organic matter and the release of nutrients [36,41]. However, the increase in the trend of the soil EOC and DOC was not significant after biochar application (Table 2). The reason for this may be that biochar improves soil porosity and aggregation, enhances soil water and air permeability, stimulates soil biological activity, and increases the soil mineralization rate [37]. Furthermore, many scholars have shown that the soil DOC can be accumulated due to the decomposition process of soil animals and plants or microbial residues and can also be reduced due to microbial consumption [42]. The EOC is mainly derived from crop roots, plant residues, and soil microbial residues. The interaction between biochar and the total-N fertilizer (CN treatments) slowed the continuous development trend of the EOC. This may be due to the fact that organic fertilizers provide a source of nutrients for microorganisms, stimulate microbial activity, increase soil respiration intensity, and soil EOC is gradually consumed and mineralized by soil microorganisms, thus reducing the soil EOC/TOC ratio [43]. The lower the soil EOC/TOC, the greater the soil carbon stability [44]. The MBC and MBN increased at a faster rate in this study than the EOC and DOC (Table 2); thus, we concluded that the MBC and MBN were more susceptible to the soil charcoal and N fertilizer additions [45]. Biochar had little effect on MBC without N fertilizer, while in the case of N fertilizer matching, biochar application was highly significant and positively correlated with the increase in the soil MBC (*p* < 0.01) up to 44.65%. The half-N fertilizer treatments (CN1/2 treatments) had a greater impact on the soil MBC, possibly because N fertilizer provides a source of nutrients for microorganisms, stimulates their activity, promotes the carbon consumption of microorganisms, and decreases the ratio of the soil EOC/TOC [43,46]. Soil MBN did not change much or even tend to decrease in the total-N fertilizer treatment (CN treatments), either because of the slow and intricate response of soil microbial biomass to N application or because there was no significant microbial N fixation after biochar application.

### 4.3. The Biochar Effects on the Activity of Enzymes

The mineralization of SOM is essential and is the process where carbon, N, and other nutrients are changed from an organic form to an inorganic one with the help of microorganisms [37]. Enzymes in the soil can stimulate the degradation of SOM and supply useful dissolved chemicals for development and metabolism in the soil [47]. Biochar can mitigate the negative effects of soil structure and function degradation on soil enzyme activity, particularly when applied in conjunction with nitrogen fertilizer, according to the findings of Song [6]. Lammirato [3] investigated the effect of activated carbon on the extracellular enzyme reaction of cellulose degradation, and the strong adsorption characteristics of biochar were mentioned. Therefore, the increase in enzyme activity may be due to activated carbon particles inhibiting the contact between the substrate and enzyme and the enzyme and substrate adsorbing onto different particles, thus avoiding enzymolysis in the adsorption process. However, Dempster [48] introduced jarrah biochar to coarse soil and discovered that biochar hindered the decomposition of soil organic matter and N mineralization, leading to a decrease in the activity of the soil microbial population. This also occurred in the present study, where urease and cellulase showed negative correlations with the MBC and MBN, respectively. Zimmerman [49] explained this phenomenon: when the soil has a higher biochar and organic matter content, the soil carbon mineralization is stronger. Or maybe the promoting effect of biochar on soil carbon mineralization does not affect its carbon sequestration effect. Principal component analysis showed that the activities of urease and sucrase showed significant positive correlations with the TOC and TN (*p* < 0.05), respectively. This suggests that biochar can provide direct nutritional value to microorganisms or can help to improve nutrient use efficiency [50].

The improvement in soil enzyme activity was more stable with the combined application treatment compared with biochar application alone. This might be due to the increased N sources that N fertilization offered for microbial decomposition and utilization of organic materials, and the soil carbon to N ratio increased, which enhanced the soil biochemical reactions and promoted material cycling, thus increasing the soil enzyme activity [47,51]. Therefore, the activity of soil enzymes was kept in check by biochar, but the addition of nitrogen was also a significant element that influenced soil enzyme activity. However, excessive N fertilization restricted the enhancement of sucrase activity.

### 4.4. The Biochar Effect on Maize Yield

Our study found that the yield of maize with the same management measures showed a large regional difference after the addition of different amounts of biochar and combined application with N fertilizer [52]. After continuous tillage in the CK treatment, there was no external carbon input in the soil, the yield was relatively low, the residual stubble of the corn crops was especially low, the number of organic materials that were returned to the soil was less, and the content of the SOC decreased year on year [53]. In addition, our research revealed that biochar greatly boosts grain production and yield stability. Since the mid-1990s, the soil and fertilizer station in Tonghua City, Jilin Province, has carried out a study on reducing the N application for corn and has achieved obvious results [54,55,56]. Using dark brown soil, alluvial soil, and black soil as examples, the yield of reduced N application was found to increase by up to 10% compared to conventional N application [57,58]. At the Lausanne Experimental Station in the UK, when the rate of N application was reduced to 150 kg·hm^2^ and 180 kg·hm^2^, the maize yield was still the highest [59]. In this study, compared with the control treatment, the combination of biochar and reduced nitrogen fertilizer had obvious advantages in improving grain quality and yield. The corn grain yield of the C3N1/2 treatment was the highest, with an increase of 49.13%. The amount of biochar and the SOC content had a substantial positive connection. Under the treatment of the combined application of biochar and N fertilizer, the level of the SOC increased significantly by 20.08–35.58%. This conclusion was also supported by Xu [60], who proposed that the effect of an enhanced crop yield was mostly explained by soil fertility and that the change in wheat yield was positively linked with the SOC and TN contents. To improve the shortage of carbon and N in the soil, the use of biochar and N fertilizer has the potential to increase soil fertility and supply additional nutrients for crop growth [61,62]. The gradual increase in enzyme activity also accelerated the soil nutrient conversion cycle, thus improving soil fertility and productivity [63].

Biochar is expected to increase soil enzyme activity, accelerate carbon humification, and promote nutrient release [64]. RDA showed that the increase in maize yield was attributed to the improvement of soil carbon and nitrogen concentrations and microbial activity (Figure 4). Therefore, it is necessary to apply biochar and nitrogen fertilizer to degraded black soils to improve soil fertility and create a favorable environment for microorganisms to survive.

## 5. Conclusions

In our study, we found a positive effect of both biochar and N fertilizer on soil C and N nutrients. Biochar significantly increased C-pool saturation by 35.18% compared to the control. There was a synergistic effect of biochar with nitrogen fertilizer to increase soil N, with a maximum increase of 56.18% in TN. The PCA showed that soil nutrient content was the main driver of enhanced soil enzyme activity. The input of exogenous organic matter promoted the decomposition and conversion of organic matter by soil enzymes and improved the soil carbon and nitrogen cycles. Significant correlations (*p* < 0.05) were found between soil organic carbon, total nitrogen content, and maize yield. Biochar-blend N fertilizer was more effective in improving maize yield than biochar alone. The best improvement was achieved with C3N1/2 treatment, with a 50.97% yield increase. In summary, rational application is the best strategy to increase soil organic carbon stocks, reduce N loss, optimize soil microstructure, and promote sustainable agricultural production.

## Figures and Tables

**Table 1:** Application rates of biochar and nitrogen fertilizer in different treatments.

Treatment	C (Mg·ha^−1^)	N (kg N ha^−1^)
CK	0	0
C1	9.8	0
C2	19.6	0
C3	29.4	0
C1N1/2	9.8	300
C2N1/2	19.6	300
C3N1/2	29.4	300
C1N	9.8	600
C2N	19.6	600
C3N	29.4	600

**Table 2:** Soil active organic carbon fraction under different treatments.

Treatments	EOC (g·kg^−1^)	DOC (mg·kg^−1^)	MBC (mg·kg^−1^)	MBN (mg·kg^−1^)	MBC/MBN
CK	3.50 abc	161.21 b	142.22 ab	18.08 a	9.79 a
C1	4.00 abc	175.79 ab	149.66 ab	21.06 a	10.03 a
C2	3.62 abc	167.75 ab	165.88 ab	15.46 ab	10.73 a
C3	3.02 bc	176.98 ab	202.08 a	16.48 ab	8.88 a
C1N1/2	4.07 ab	164.26 ab	170.63 ab	20.96 a	11.95 a
C2N1/2	3.87 abc	174.92 ab	185.23 ab	18.50 a	10.06 a
C3N1/2	4.61 a	179.03 ab	205.72 a	14.48 ab	9.81 a
C1N	2.67 c	169.29 ab	105.75 b	13.20 ab	19.88 a
C2N	2.85 bc	181.77 ab	127.00 ab	8.48 b	19.80 a
C3N	3.59 abc	184.87 a	140.37 ab	8.36 b	16.88 a

Different letters indicate statistically significant (*p* < 0.05) differences between treatments

**Table 3:** The ratio of activated organic carbon fraction to TOC under different treatments.

Treatments	EOC/TOC (%)	DOC/TOC (%)	MBC/TOC (%)
CK	46.22 ab	2.17 a	2.45 a
C1	48.58 a	2.12 ab	2.45 a
C2	40.34 ab	1.81 c	1.79 abc
C3	30.09 b	1.77 c	1.42 bc
C1N1/2	44.17 ab	1.84 c	1.93 abc
C2N1/2	42.07 ab	1.90 bc	2.01 abc
C3N1/2	49.08 a	1.91 bc	2.19 ab
C1N	29.43 b	1.79 c	1.17 c
C2N	30.15 b	1.92 bc	1.34 bc
C3N	35.98 ab	1.84 c	1.73 abc

Different letters indicate statistically significant (*p* < 0.05) differences between treatments

**Table 4:** Agronomic traits of wheat under different treatments.

Treatments	Ear Length (cm)	Panicle Weight (g)	Seeds/Ear	1000-Weight (g)	Grain Water Content (%)	Yield (kg/hm^2^)
CK	17.10 b	176.67 c	495.34 b	386.60 ab	0.18 a	9322.49 c
C1	18.24 b	252.27 bc	560.44 ab	372.30 b	0.16 ab	9423.06 c
C2	18.40 ab	346.88 ab	537.89 ab	432.00 ab	0.13 abc	10,066.11 bc
C3	19.41 b	272.22 bc	588.66 ab	448.67 ab	0.13 bc	11,436.93 abc
C1N1/2	20.75 ab	326.32 ab	603.73 ab	443.20 ab	0.16 bc	13,749.08 a
C2N1/2	21.42 ab	336.84 ab	648.11 a	440.00 ab	0.11 c	13,144.62 ab
C3N1/2	21.04 a	277.78 ab	647.06 a	419.20 ab	0.14 bc	14,052.62 a
C1N	20.46 ab	270.37 bc	603.42 ab	446.00 a	0.13 c	12,697.08 abc
C2N	20.88 ab	321.30 ab	611.33 ab	478.50 a	0.13 bc	12,366.94 abc
C3N	20.15 ab	301.82 ab	618.51 ab	447.10 ab	0.14 c	12,296.25 abc

Different letters indicate statistically significant (*p* < 0.05) differences between treatments

**Table 5:** Principal component evaluation value and comprehensive evaluation value.

Treatments	F1	F2	F3	F	Rank
CK	−3.88	1.81	−0.52	−1.30	10
C1	−1.05	1.86	−0.10	0.17	5
C2	−0.95	0.60	−0.84	−0.34	7
C3	0.95	−0.32	−1.39	0.18	4
C1N1/2	−0.63	0.00	1.61	−0.12	6
C2N1/2	0.93	0.72	0.76	0.83	3
C3N1/2	3.04	1.86	1.02	2.35	1
C1N	−1.65	−3.49	1.06	−1.03	9
C2N	0.39	−2.60	−0.79	−0.89	8
C3N	2.85	−0.44	−0.81	1.15	2

## Data Availability

The data are not publicly available. A deidentified dataset can be obtained by contacting the correspondence author.

## References

[1] Islam M.U., Jiang F., Guo Z., Peng X. (2021). Does biochar application improve soil aggregation? A meta-analysis. Soil Tillage Res..

[2] Joseph U.E., Toluwase A.O., Kehinde E.O., Omasan E.E., Tolulope A.Y., George O.O., Zhao C., Hongyan W. (2020). Effect of biochar on soil structure and storage of soil organic carbon and nitrogen in the aggregate fractions of an Albic soil. Arch. Agron. Soil Sci..

[3] Lammirato C., Miltner A., Kaestner M. (2011). Effects of wood char and activated carbon on the hydrolysis of cellobiose by beta-glucosidase from Aspergillus niger. Soil Biol. Biochem..

[4] Glaser B., Balashov E., Haumaier L., Guggenberger G., Zech W. (2000). Black carbon in density fractions of anthropogenic soils of the Brazilian Amazon region. Org. Geochem..

[5] Hu L., Li S., Li K., Huang H., Wan W., Huang Q., Li Q., Li Y., Deng H., He T. (2020). Effects of Two Types of Straw Biochar on the Mineralization of Soil Organic Carbon in Farmland. Sustainability.

[6] Song D., Chen L., Zhang S., Zheng Q., Ullah S., Zhou W., Wang X. (2020). Combined biochar and nitrogen fertilizer change soil enzyme and microbial activities in a 2-year field trial. Eur. J. Soil Biol..

[7] Luo M., Tian D., Gao M., Huang R. (2018). Response of organic carbon activity fraction to biochar application in purple soils. Environ Sci..

[8] Demisie W., Liu Z., Zhang M. (2014). Effect of biochar on carbon fractions and enzyme activity of red soil. Catena.

[9] Shang J., Geng Z.C., Chen X.X., Zhao J., Geng R., Wang S. (2015). Effects of biochar application on organic carbon, nitrogen and their fractions in dry farmland soils. J. Agro-Environ. Sci..

[10] Liu C.H., Chu W., Li H., Boyd S.A., Teppen B.J., Mao J., Lehmann J., Zhang W. (2019). Quantification and characterization of dissolved organic carbon from biochars. Geoderma.

[11] Pan Q.-L., Song T., Chen K., Xu X.-N., Peng J., Zhang X.-M., Wang Y., Han X.-R. (2016). Effect of continuous 6-year application of biochar and charcoal-based fertilizer on the biological activity of brown soil. J. North China Agric..

[12] Dominik A., Franz Z., Simone J., Lisa J., Georg P., Bernhard W., Franz R., Florian F., Georg D., Sophie Z.B. (2018). Activated biochar alters activities of carbon and nitrogen acquiring soil enzymes. Pedobiologia.

[13] Aziz H., Wang X., Murtaza G., Ashar A., Hussain S., Abid M., Murtaza B., Saleem M.H., Fiaz S., Ali S. (2021). Evaluation of Compost and Biochar to Mitigate Chlorpyrifos Pollution in Soil and Their Effect on Soil Enzyme Dynamics. Sustainability.

[14] Amoakwah E., Arthur E., Frimpong K.A., Lorenz N., Rahman M.A., Nziguheba G., Islam K.R. (2022). Biochar amendment impacts on microbial community structures and biological and enzyme activities in a weathered tropical sandy loam. Appl. Soil Ecol..

[15] Ali I., Ullah S., He L., Zhao Q., Iqbal A., Wei S., Shah T., Ali N., Bo Y., Adnan M. (2020). Combined application of biochar and nitrogen fertilizer improves rice yield, microbial activity and N-metabolism in a pot experiment. PeerJ.

[16] Mete F.Z., Mia S., Dijkstra F.A., Abuyusuf M., Iqbal Hossain A.S.M. (2015). Synergistic Effects of Biochar and NPK Fertilizer on Soybean Yield in an Alkaline Soil. Pedosphere.

[17] Kumar D., Patel R.A., Ramani V.P., Rathod S.V. (2021). Evaluating Precision Nitrogen Management Practices in Terms of Yield, Nitrogen Use Efficiency and Nitrogen Loss Reduction in Maize Crop Under Indian Conditions. Int. J. Plant Prod..

[18] Shi D.-L., Wang X.-L., Duan J.-J., Liu A.-K., Luo A.-H., Li R.-D., Hou Z.-F. (2020). Effects of chemical N fertilizer reduction combined with biochar application on soil organic carbon active components and mineralization in paddy fields of yellow soil. J. Appl. Ecol..

[19] Li G.H., Cheng G.G., Lu W.P., Lu D.L. (2021). Differences of yield and nitrogen use efficiency under different applications of slow release fertilizer in spring maize. J. Integr. Agric..

[20] Plaza C., Giannetta B., Fernández J.M., López-De-Sá E.G., Polo A., Gascó G., Méndez A., Zaccone C. (2016). Response of different soil organic matter pools to biochar and organic fertilizers. Agric. Ecosyst. Environ..

[21] Grunwald D., Kaiser M., Ludwig B. (2016). Effect of biochar and organic fertilizers on C mineralization and macro-aggregate dynamics under different incubation temperatures. Soil Tillage Res..

[22] Hou Y.P., Yin C.X., Kong L.L. (2016). Effect of nitrogen fertilizer application on yield, agronomic efficiency and nitrogen balance of spring maize. Soil Fertil. Sci. China.

[23] Abukari A. (2014). Effect of Rice Husk Biochar on Maize Productivity in the Guinea Savannah Zone of Ghana.

[24] An N., Zhang L., Liu Y., Shen S., Li N., Wu Z., Yang J., Han W., Han X. (2022). Biochar application with reduced chemical fertilizers improves soil pore structure and rice productivity. Chemosphere.

[25] Chen J., Chen D., Xu Q., Fuhrmann J.J., Li L., Pan G., Li Y., Qin H., Liang C., Sun X. (2019). Organic carbon quality, composition of main microbial groups, enzyme activities, and temperature sensitivity of soil respiration of an acid paddy soil treated with biochar. Biol. Fertil. Soils.

[26] Azeem M., Hayat R., Hussain Q., Tahir M.I., Imran M., Abbas Z., Sajid M., Latif A. (2019). Effects of biochar and NPK on soil microbial biomass and enzyme activity during 2 years of application in the arid region. Arab. J. Geosci..

[27] Zhang Y., Li X., Gregorich E.G., McLaughlin N.B., Zhang X., Guo Y.F., Gao Y., Liang A. (2020). Tillage and cropping effects on soil organic carbon: Biodegradation and storage in density and size fractions. Eur. J. Soil Sci..

[28] Walkley A., Black I.A. (1934). An examination of the Degtjareff method for determining soil organic matter, and a proposed modification of the chromic acid titration method. Soil Sci..

[29] Lin L., Gao Z., Liu X. (2020). Estimation of soil total nitrogen using the synthetic color learning machine (SCLM) method and hyperspectral data. Geoderma.

[30] Lian P.K., Xu Z.-C., Meng L.M., Liu B.Q., Zhai X., Chen X., Huang H.G. (2016). Comparison of carbon and nitrogen metabolism of flue-cured tobacco in different altitudes in Wumeng tobacco-growing area of Guizhou. J. Plant Nutr. Fertil..

[31] Yang P., Liu Y., Zhang J. Determination of cellulase activity by glucokinase method. Proceedings of the 10th All Army Conference on Laboratory Medicine.

[32] Vilar R.P., Ikuma K. (2021). Adsorption of urease as part of a complex protein mixture onto soil and its implications for enzymatic activity. Biochem. Eng. J..

[33] Meng F., Yu X., Wang Z., Hu S., Sun J., Qing G., Qu J., Gao J. (2020). Effect of biochar with nitrogen fertilizer on soil physical properties and yield of spring maize. Maize Sci..

[34] Zhang S., Cui J., Wu H., Zheng Q., Song D., Wang X., Zhang S. (2021). Organic carbon, total nitrogen, and microbial community distributions within aggregates of calcareous soil treated with biochar. Agric. Ecosyst. Environ..

[35] Xiu L., Zhang W., Sun Y., Wu D., Meng J., Chen W. (2019). Effects of biochar and straw returning on the key cultivation limitations of Albic soil and soybean growth over 2 years. Catena.

[36] Zhang Y., Li X., Gregorich E.G., McLaughlin N.B., Zhang X., Guo Y., Liang A., Fan R., Sun B. (2018). No-tillage with continuous maize cropping enhances soil aggregation and organic carbon storage in Northeast China. Geoderma.

[37] Lu W., Zhang H. (2015). Respose of biochar induced carbon mineralization priming effects to additional nitrogen in a sandy loam soil. Appl. Soil Ecol..

[38] Wu X.-M., Yang X.-Y., Lin Y.-Y., Dai Y., Cao L., Dong W.-X., Liu Z.-W. (2021). Response of Humification of *Robinia pseudoacacia* and *Pinus tabulaeformis* Litter to Climate Temperature and Humidity Changes. Acta Agrestia Sin..

[39] Li J., Sang C., Yang J., Qu L., Wang C. (2021). Stoichiometric imbalance and microbial community regulate microbial elements use efficiencies under nitrogen addition. Soil Biol. Biochem..

[40] Devereux R.C., Sturrock C.J., Mooney S.J. (2012). The effects of biochar on soil physical properties and winter wheat growth. Earth Environ. Sci. Trans. R. Soc. Edinb..

[41] Zhao C., Zhang Y., Liu X., Ma X., Meng Y., Li X., Quan X., Shan J., Zhao W., Wang H. (2020). Comparing the Effects of Biochar and Straw Amendment on Soil Carbon Pools and Bacterial Community Structure in Degraded Soil. J. Soil Sci. Plant Nutr..

[42] Bu D., Zhou D.-Y., Ge Z.-W., Wang G.-B., Zhang W.-W., Peng S., Ruan H.-H., Cao G.-H. (2015). Effect of methane application on soil reactive organic carbon in poplar plantations in northern Jiangsu Province. J. Ecol..

[43] Duan Y., Yang J., Song Y., Chen F., Li X., Awasthi M.K., Li H., Zhang L. (2021). Clean technology for biochar and organic waste recycling, and utilization in apple orchard. Chemosphere.

[44] Lai J.C., Gao M., Tian D., Huang R., Xu G.X. (2018). Effect of straw and biochar on soil organic carbon and its active fraction. J. Grassl. Sci..

[45] Xu H.Q., Zhang J.E., Feng L.F., Quan G.M., Qin Z. (2009). Effects of different land use patterns on microbial biomass carbon and nitrogen in Guangdong Province. Acta Ecol. Sin..

[46] Gao Y., Shao S., Zhang W., Wang L., He H., Zhang X. (2020). Response of organic matter content and fraction to no-till straw mulch in black soils of Northeast China. J. Dalian Jiaotong Univ..

[47] Liu S., Zhang Y., Zong Y., Hu Z., Wu S., Zhou J., Jin Y., Zou J. (2015). Response of soil carbon dioxide fluxes, soil organic carbon and microbial biomass carbon to biochar amendment: A meta-analysis. Gcb Bioenergy.

[48] Dempster D.N., Gleeson D., Solaiman Z., Jones D.L., Murphy D. (2012). Decreased soil microbial biomass and nitrogen mineralisation with Eucalyptus biochar addition to a coarse textured soil. Plant Soil.

[49] Zimmerman A.R., Ouyang L. (2019). Priming of pyrogenic C (biochar) mineralization by dissolved organic matter and vice versa. Soil Biol. Biochem..

[50] Luo L., Gu J.-D. (2016). Alteration of extracellular enzyme activity and microbial abundance by biochar addition: Implication for carbon sequestration in subtropical mangrove sediment. J. Environ. Manag..

[51] Jia X., Zhong Y., Liu J., Zhu G., Shangguan Z., Yan W. (2020). Effects of nitrogen enrichment on soil microbial characteristics: From biomass to enzyme activities. Geoderma.

[52] Hale S.E., Nurida N.L., Jubaedah, Mulder J., Sørmo E., Silvani L., Abiven S., Joseph S., Taherymoosavi S., Cornelissen G. (2020). The effect of biochar, lime and ash on maize yield in a long-term field trial in a Ultisol in the humid tropics. Sci. Total. Environ..

[53] Dikgwatlhe S.B., Chen Z.-D., Lal R., Zhang H.-L., Chen F. (2014). Changes in soil organic carbon and nitrogen as affected by tillage and residue management under wheat–maize cropping system in the North China Plain. Soil Tillage Res..

[54] Zhang X., Zhan Q., Li W. (2000). Study on the effect of fertilizer on maize yield increase and its utilization rate on black soil in Jilin. Agric. Mod. Res..

[55] Chen Z., Sui B., Zhao X., Wang H., Zhao L. (2018). Study on the effect of nitrogen fertilizer reduction in maize in black soil areas of Jilin Province. Maize Sci..

[56] Bai Y.X. (2019). High-yielding cultivation and pest control technology of high-quality maize in Tonghua, Jilin. Agric. Eng. Technol..

[57] Li M.Y., Wang Y., Ji F., Wang K.S., Gao S.Q., Wang L., Cao Z.Y., Zhang J.J., Wang L.C. (2016). Studies on Maize Yield and Nitrogen Use Efficiency on Major Types of Arable Soils in Jilin Province. J. Northeast Agric. Sci..

[58] Zhou Y., Xie J., Li L., Wang L., Luo Z., Wang J. (2021). Regulation of long-term no-till and N fertilizer reduction on yield and carbon emission of all-membrane double-monopoly furrow-sown maize. Chin. Agric. Sci..

[59] Chen P., Wu L., Zheng M., Cui J., Cheng S., Wu D., Hu L. The simulating test of the environmental capacity of biogas fertilizer utilization on wheat and corn planting system. Proceedings of the International Conference on Materials for Renewable Energy & Environment.

[60] Xu Z. (2019). Long-term effects of tillage and straw management on soil organic carbon, crop yield, and yield stability in a wheat-maize system. Field Crops Res..

[61] Dong D., Wang C., Van Zwieten L., Wang H., Jiang P., Zhou M., Wu W. (2020). An effective biochar-based slow-release fertilizer for reducing nitrogen loss in paddy fields. J. Soils Sediments.

[62] Liu Z., Tang J., Ren X., Schaeffer S.M. (2021). Effects of phosphorus modified nZVI-biochar composite on emission of greenhouse gases and changes of microbial community in soil. Environ. Pollut..

[63] Wang H., Shi M., Xu Y., Lu H., Xu W., Guan H. (2021). Effect of biochar and organic fertilizer on the enzyme activity and crop yield of red soil in arable land. J. Yunnan Norm. Univ. Nat. Sci. Ed..

[64] Sun Z., Bruun E.W., Arthur E., de Jonge L.W., Moldrup P., Hauggaard-Nielsen H., Elsgaard L. (2014). Effects of biochar on aerobic processes, enzyme activity, and crop yields in two sandy loam soils. Biol. Fertil. Soils.

